# Technological advances in the diagnosis and management of inherited optic neuropathies

**DOI:** 10.3389/fneur.2025.1609033

**Published:** 2025-07-25

**Authors:** John O. T. Britton, Patrick Yu-Wai-Man, Benson S. Chen

**Affiliations:** ^1^John van Geest Centre for Brain Repair and MRC Mitochondrial Biology Unit, Department of Clinical Neurosciences, University of Cambridge, Cambridge, United Kingdom; ^2^Cambridge Eye Unit, Addenbrooke’s Hospital, Cambridge University Hospitals, Cambridge, United Kingdom; ^3^Moorfields Eye Hospital NHS Foundation Trust, London, United Kingdom; ^4^Institute of Ophthalmology, University College London, London, United Kingdom; ^5^Department of Medicine, University of Auckland, Auckland, New Zealand

**Keywords:** optic atrophy, hereditary, leber, autosomal dominant, retinal ganglion cells

## Abstract

Preferential degeneration of retinal ganglion cells (RGCs) is a defining feature of the inherited optic neuropathies (IONs), a group of monogenic eye diseases predominately comprising Leber hereditary optic neuropathy (LHON) and autosomal dominant optic atrophy (DOA). Their pathogenesis is characterised by mitochondrial dysfunction, which causes loss of RGCs leading to irreversible vision loss. Although currently incurable, there are several emerging therapeutic avenues encompassing gene therapies, precision medicine strategies and neuroprotection. These are underscored by recent technological advances such as next-generation sequencing and improved disease modelling. In this review, we discuss these advances and the impact these will have on future diagnostic and treatment capabilities. We first focus on the clinical presentation and pathogenic mechanisms of LHON and DOA, followed by a discussion of emerging technology to facilitate diagnosis and treatment. We highlight the current unmet clinical demand of IONs, and the promise of current and future research developments.

## Introduction

Retinal ganglion cells (RGCs) are an inner retinal cell type responsible for transmission of visual stimuli to the central nervous system. RGC axons combine to form the retinal nerve fiber layer (RNFL), which converges at the optic disc to form the optic nerve. The optic nerve continues posteriorly reaching the optic chiasm. The crossing axons form the optic tracts, which synapse at the lateral geniculate nucleus of the thalamus. The preferential loss of RGCs is a defining feature of the inherited optic neuropathies (IONs): diseases of the optic nerve which predominately comprise Leber hereditary optic neuropathy (LHON; OMIM #535000) and autosomal dominant optic atrophy (DOA; OMIM #165500). Despite disparate disease mechanisms, both conditions show selective vulnerability of the papillomacular bundle—a portion of the retina formed by RGC axons originating from the macula—and thinning of the RNFL. This progresses with increasing age and causes irreversible vision loss (1).

Ubiquitous expression of the genes implicated in LHON and DOA is puzzling given the effect mutations have on RGCs specifically. Fully defining this pathological process remains unresolved. Proposed mechanisms reflect the inherent structure of RGCs, which are unmyelinated to facilitate optical transparency. Unable to re-establish membrane potentials as efficiently as myelinated axons, RGCs have much greater metabolic demands which causes a corresponding strain on mitochondria. This increased metabolic requirement makes RGCs vulnerable to mitochondrial dysfunction and leads to apoptosis when unable to meet energy requirements (2, 3).

Human RGCs are categorised based on morphology into 18 subtypes, with midget and parasol RCGs comprising ~80% of RGC population (4). These subtypes degenerate in IONs to cause impaired sensitivity to spatial frequencies and colour discrimination. Intrinsically photosensitive RGCs (ipRGCs) are a subtype that express the photopigment melanopsin. Although only comprising ~1% of the RGC population, ipRGCs are crucial to physiology of the pupillary light reflex and light responses underlying the circadian rhythm. Interestingly, investigations have demonstrated relative preservation of ipRGCs in IONs both functionally (5) and histologically (6). The mechanism of this relative preservation in IONs is unclear, but this peculiarity may prove useful as a possible biomarker and provide clues to developing neuroprotective strategies.

Greater access to genetic testing and the integration of genetic counselling in clinical ophthalmic settings is helping to increase awareness of IONs alongside other rare genetic causes of vision loss. Wider access to next-generation high throughput sequencing, and the improvement of disease modelling techniques are also furthering the diagnostic yield of IONs. In this review, we will focus on these technological advancements, including their influence on improving the diagnosis and management of IONs, which promises to ameliorate an urgent clinical need for these blinding disorders. We firstly summarise the clinical presentation and pathogenetic mechanisms that underpin LHON and DOA, and current treatment strategies. We then discuss technological advancements including the development of disease models and genetic technologies that underpin emerging therapeutic options for LHON and DOA.

## Leber hereditary optic neuropathy (LHON)

LHON is a maternally-inherited mitochondrial disease which shows a preponderance for men, who are three-to-five times more likely to be affected than women among those at risk of the disease (7). Sex does not appear to affect severity or timing of disease. LHON usually follows a sequential course in which one eye develops painless vision loss and the fellow eye is subsequently affected, almost always within 1 year (8). However, ~25% of patients present with bilateral simultaneous optic nerve involvement. Vision loss is severe in LHON, with most patients experiencing a significant reduction in vision-related quality of life (9, 10).

The clinical course of LHON may be grouped according to the time after onset of vision loss (11). In the subacute stage, 0–6 months after onset of vision loss, there is rapid deterioration with an expanding central or cecocentral scotoma. Visual acuity stabilises in the dynamic stage, 6–12 months after disease onset. Patients then reach the chronic stage after 12 months, in which visual function does not deteriorate any further and a minority of patients (particularly those with the m.14484T>C mutation) may exhibit spontaneous partial visual improvement (12).

Three common mitochondrial DNA (mtDNA) mutations account for ~90% of LHON cases, comprising m.11778G>A, m.3460G>A and m.14484T>C (13). These mutations affect *MT-ND1*, *MT-ND4* and *MT-ND6* genes respectively, resulting in defective ND1, ND4 and ND6 subunits, which are critical subunits of complex I of the mitochondrial electron transport chain (2). Altered mitochondrial respiration results in compromised ATP production and the production of reactive oxygen species (ROS), with RGC degeneration due to a bioenergetic shortfall. Clinical findings differ depending on disease stage: initially the optic disc may appear hyperaemic with pseudo-oedema, eventually progressing towards optic atrophy with a pale optic disc and corresponding thinning of the RNFL, which can be quantified by optical coherence tomography (OCT). Patients may demonstrate extraocular neurological abnormalities, including peripheral neuropathy, a postural tremor and MS-like features—the combined presentation of LHON with MS-like features has been referred to as Harding’s disease.

The visual prognosis is partly determined by the underlying LHON mtDNA variant (genotype). However, the genotype does not fully explain the clinical presentation (phenotype). For example, patients exhibit incomplete penetrance characterised by an inconsistent pattern of vision loss in carriers who harbour a pathogenic LHON mutation. A *two-hit hypothesis* likely underscores this, in which environmental triggers, such as smoking, impair mitochondrial function resulting in a tipping point. This leads to a bioenergetic crisis with activation of cellular apoptosis and an alteration in the balance between mitochondrial biogenesis and mitophagy (14). Disease penetrance is also influenced, in part, by mitochondrial haplogroup. Individuals with the mitochondrial haplogroup U may have a lower risk of developing LHON-related vision loss (15).

Incomplete penetrance in LHON is further complicated by the proportion of mitochondria with LHON mtDNA variants, termed heteroplasmy or homoplasmy, and the bearing this has on mutation carriers developing the phenotype. The commonest mutations causing LHON are usually homoplasmic. However, heteroplasmic mothers must be carefully counselled regarding the risks of transmission to offspring. This is due to the presence of a *genetic bottleneck* in early embryological development, which causes generational shifts in mitochondrial allele frequencies and complicates accurate predictions of heritability (16, 17).

Spontaneous improvement in vision is observed in a minority of LHON patients. Often described as ‘recovery’, it is important to note this is a misnomer and most LHON patients will remain significantly visually impaired, with poor visual acuity and quality of life (9, 10). The rate of spontaneous improvement in LHON is not clearly defined, although estimated to be 11.3% in patients with the m.11778G>A mutation, and occurs most frequently in patients with the m.14484T>C mutation for whom rate of spontaneous improvement has been reported at 64% (11, 12, 18, 19). Factors influencing this include patients with a younger age of onset, milder visual impairment at nadir and a larger optic disc (8, 20–22). The recovery may occur progressively or suddenly, typically with the reappearance of discrete areas of vision within the scotoma (termed *fenestration*). These complicating factors make predicting outcomes and genetic counselling in patients with LHON particularly challenging. Preclinically, they underscore the importance of rigorous prospective natural history studies to fully elucidate disease mechanisms and provide a suitable control for clinical trials. This is especially pertinent given the promise of recent therapeutic developments for treatment of LHON.

## Autosomal dominant optic atrophy (DOA)

DOA is the most common ION with an estimated minimum prevalence of 1:25,000 (23). Patients present with painless insidious vision loss, often in early childhood. Vision loss is bilateral and typically symmetrical. It follows a progressive though gradual course, initially with a central or cecocentral scotoma and dyschromatopsia (24).

Although following a slow course, the degree of vision loss displays marked inter- and intrafamilial variability, ranging from normal, or mildly reduced, to detection of hand movements. Fundus examination may demonstrate pallor of the optic discs, particularly temporally, and OCT will demonstrate reduced RNFL thickness. This especially affects the papillomacular bundle and is accompanied by thinning of the retinal ganglion cell layer, observed on macular OCT (25). A subset of patients exhibit a constellation of neurological symptoms including sensorineural hearing loss and peripheral neuropathy—so-called *DOA plus* (26). The causative gene in over 60% of cases is *OPA1*, located on chromosome 3q28-29 (27). Other implicated genes are emerging with the advent of improved sequencing technologies, especially in cases of *DOA plus* [(28, 29), for a thorough review of causative genes in DOA, see (24)].

*OPA1* is a large nuclear gene consisting of 31 exons, three of which (exons 4, 4b and 5b) facilitate alternative splicing variants to encode eight isoforms of the protein. The latter is a ubiquitously expressed dynamin-like GTPase that catalyses the conversion of guanosine triphosphate (GTP) into guanosine diphosphate (GDP). This protein is imported into the inner mitochondrial membrane via an N-terminal mitochondrial targeting sequence (MTS) (30). It exists in both long and short-forms, termed L-OPA1 and S-OPA1 respectively, to regulate mitochondrial mechanisms (31). These include mitochondrial fusion, maintenance of cristae and mitochondrial bioenergetics (32). Thus, DOA is a nuclear-encoded mitochondrial optic neuropathy.

Alongside the MTS, the OPA1 protein consists of further distinct components relating to its function. Over 500 *OPA1* variants are thought to cause DOA^1^ and cluster in these different regions across the coding sequence (28). A reported two thirds are mutations located in the protein’s GTPase domain (29) and give rise to haploinsufficiency—the process by which a variant results in loss of function of the mutated allele due to a premature termination codon, causing translation of truncated OPA1 polypeptides and nonsense mediated decay (NMD). A minority of mutations are located upstream to the GTPase domain, and very few disease-causing variants have been reported in the region of the gene’s alternatively spliced exons (29, 33). Other variant subtypes include altered splicing, frameshift mutations, nonsense mutations and deletions (28). Dominant-negative mutations are largely caused by missense variants, in which disruption of the wild-type (WT) gene results in the expression of mutant mRNA that escapes NMD, to produce an aberrant protein. Clinically, missense variants are more strongly associated with the *DOA plus* phenotype and a worse visual prognosis (26). Given the genetic heterogeneity of DOA, and despite recent advances in sequencing technologies, a third of patients with DOA phenotype receive no confirmed molecular diagnosis (34).

## LHON therapeutic advances

**Figure 1:**
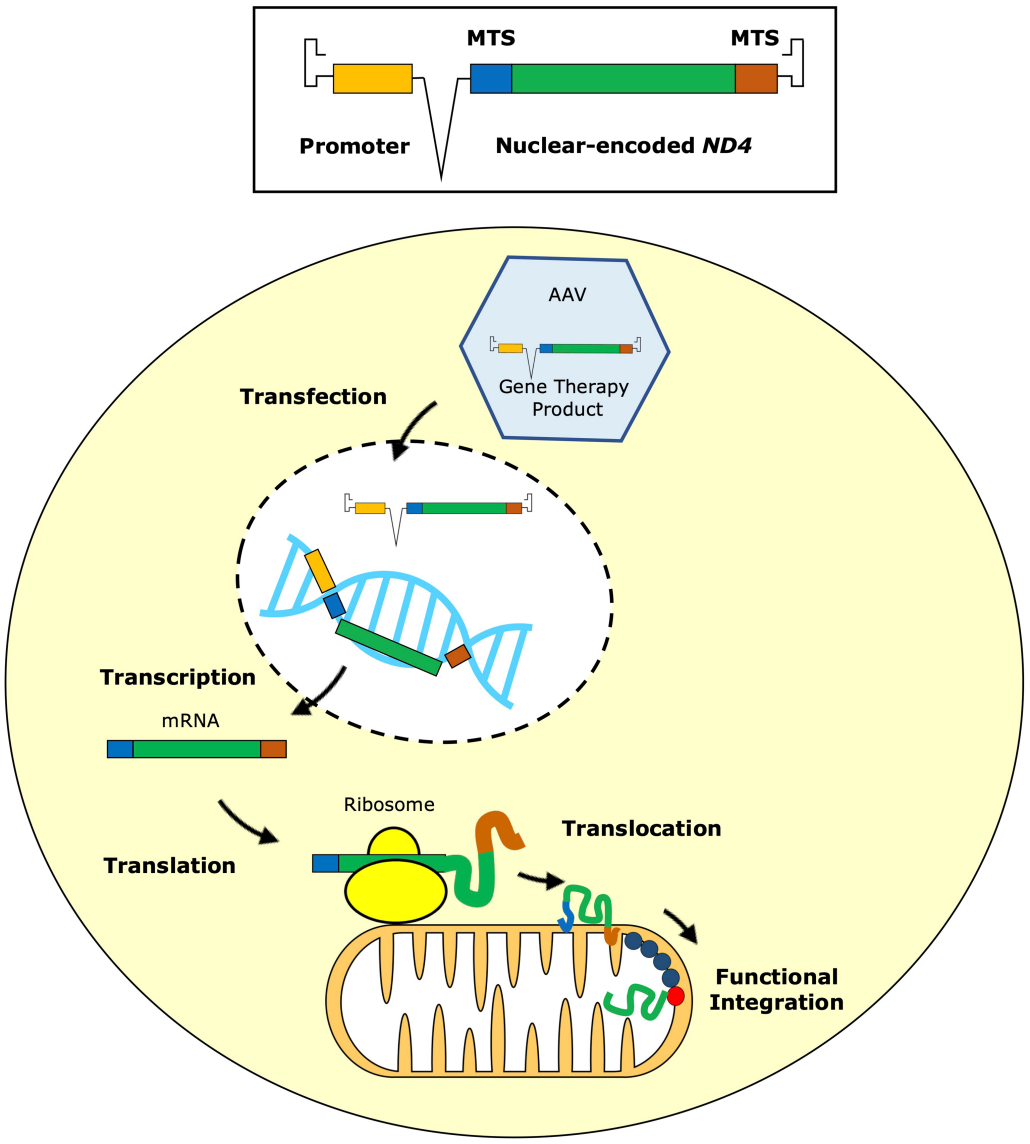
Reproduced with permission from Chen et al. (2023). The gene therapy vector contains the replacement mitochondrial *ND4* gene modified according to the nuclear code. This nuclear-encoded version of *ND4* is fused to two mitochondrial targeting sequences (MTS) and a promoter is inserted for more efficient expression. The gene therapy vector is packaged into an adeno-associated virus (AAV), usually serotype 2, which is delivered to the retinal ganglion cells by intravitreal injection. The AAV transfects the retinal ganglion cells, delivering the gene therapy product into the nucleus, where it remains in an episomal state. From here, the inserted gene sequence undergoes transcription to messenger RNA (mRNA) and, with the aid of the MTS, it is directed to mitochondrial ribosomes on the external surface of the mitochondrial outer membrane, where gene translation occurs. As it is being synthesised, the newly translated ND4 protein is translocated across the two mitochondrial membranes into the mitochondrial matrix aided by the MTS. Finally, the imported ND4 subunit undergoes post-translational folding before being integrated within complex I, restoring its function.

The eye is an attractive organ for testing new therapeutic modalities such as gene therapy. It is easily accessible, with relative immune privilege, and can be assessed both structurally and functionally. This is useful for direct delivery of intraocular gene therapy vectors and assessment for procedural or pharmacological side-effects. Difficulties arise when targeting the mitochondrial genome, as the gene therapy must account for the physical barrier imposed by the outer and inner mitochondrial membranes. Gene therapies have circumvented this with allotopic expression (35), in which an adenoviral vector (AAV) delivers a recoded version of the mtDNA gene to the nucleus where it is episomally expressed. The gene therapy construct contains an MTS, which is engineered for subsequent transport of the translated WT protein across the outer mitochondrial membrane (Figure 1).

Gene therapy for the m.11778G>A mutation in *MT-ND4* has been developed by several groups (36), with the first human trial of gene therapy conducted in 2011 in Wuhan, China (Table 1). To date, only one gene therapy product, lenadogene nolparvovec (GS010, GenSight Biologics), has been investigated as part of four multinational phase III trials with peer-reviewed published results. The construct uses a recombinant AAV to deliver a WT copy of *MT-ND4* via allotopic expression. Preclinical studies and phase III trials have demonstrated the therapy’s favourable efficacy and safety profile, including a sustained improvement in best corrected visual acuity (BCVA) with minimal adverse events based on 5 years of follow-up data (37–40). A recent meta-analysis demonstrated visual improvement superior to that observed in LHON natural history (11). Despite this success, clearly defining visual improvements in these trials has been complicated by the lack of an internal control group due to unexpected bilateral improvement in BCVA despite unilateral intravitreal injection. There remains a need for ongoing rigorous prospective natural history studies to clearly detail the clinical course of LHON, and for future clinical trials to include a true control group to help fully differentiate treatment effect from possible spontaneous visual improvement (Table 1).

**Table 1:** Summary of therapeutic options for Leber hereditary optic neuropathy (LHON) and autosomal dominant optic atrophy (DOA), including new diagnostic technologies, disease modelling and emerging treatment strategies.

Therapeutic options for Leber hereditary optic neuropathy (LHON)	
Oral	**Idebenone** Ubiquinone analogue, bypasses dysfunctional complex I in mitochondrial electron transport chain Approved by the **European Medicines Agency** for use in LHON RHODOS and RHODOS-OFU trials demonstrated efficacy in LHON patients (48–50) LEROS trial demonstrated long-term effectiveness (51)
Intravitreal gene therapy	**Lenadogene nolparvovec** (GS010, GenSight Biologics) Targets m.11778G>A *MT-ND4* pathogenic LHON mutation Employs a mitochondrial targeting sequence to cause therapeutic effect via **allotopic expression** Overcomes barrier imposed by the mitochondrial inner and outer membranes Adenoviral vector delivers a recoded version of the mitochondrial DNA gene to the nucleus, where it is episomally expressed Translated wild-type protein enters outer mitochondrial membrane via a mitochondrial targeting sequence Four multinational phase III clinical trials demonstrate a favourable efficacy and safety profile (36–39) **scAAV2-P1*ND4*v2** (Bascom Palmer Eye Institute, Florida, USA) Targets m.11778G>A *MT-ND4* pathogenic LHON mutation Pre-clinical animal models show favourable efficacy and safety profile (42) Phase I trial showed a small efficacy effect (NCT02161380) (43, 44) **rAAV2-*ND4*** (NFS-01, Neurophth Inc., Huazhong University, Wuhan, China) Targets m.11778G>A *MT-ND4* pathogenic LHON mutation Multicenter trial showed significant improvement in visual acuity and a favourable safety profile (45–47) Phase III trial results expected in 2026 (NCT03428178)
Other treatment strategies	Improving **mitochondrial function** and ameliorating retinal ganglion cell degeneration Addressing causes of **reactive oxygen species**: Filter to reduce exposure to low wavelength visible blue light (61) Consider potential drug interactions with mitochondrial bioenergetics—though it is challenging to prove a causal relationship in LHON patients **Lifestyle** modifications: Counselling regarding smoking cessation and alcohol consumption

Therapeutic options for autosomal dominant optic atrophy (DOA)	
Oral	**Idebenone** Current area of investigation in DOA Prospective phase II trial showed a small improvement in visual acuity (60) Being used ‘off-label’ in DOA by some clinicians
Intravitreal gene therapy	**STK-002** (Stoke Therapeutics) **Antisense oligonucleotide** Addresses haploinsufficiency (the predominant pathogenic mechanism in DOA) by increasing OPA1 protein expression Functions independent of specific pathogenic mutations Entering Phase I clinical trial (ISRCTN41725621—currently postponed) (55) **PYC-001** (PYC Therapeutics) Also uses antisense oligonucleotide approach Entering Phase I clinical trial (NCT06461286) (56)
Other treatment strategies	**mRNA *trans*-splicing** Emerging technology based on mRNA correction Splices mutated endogenous mRNA transcript to be replaced with a wild-type mRNA sequence (57) Addressing causes of **reactive oxygen species**, as in Leber hereditary optic neuropathy **Supplements** to upregulate mitochondrial biogenesis Vitamin B3 and NAD precursors eg nicotinamide may improve mitochondrial function Experimental benefit for mitochondrial in general, but unproven efficacy in inherited optic neuropathies more specifically

Defining new pathological processes	
Disease modelling	**Induced pluripotent stem cells (iPSCs)**: Embryonic stem cell-like cells capable of differentiating into any cell type from the three germ layers Used as a disease model to study pathological processes *in vitro* Retinal ganglion cells in the case of inherited optic neuropathies Allow an assessment of patient-derived cells with specific mutations **CRISPR-Cas9** Established method of editing nuclear-encoded gene mutations by using an endonuclease May be combined with an iPSC model to determine possible therapeutic targets
Sequencing technology	**Next-generation sequencing** Expanding our understanding of genetic determinants Promises to help answer elusive questions, such as the method of incomplete penetrance in Leber hereditary optic neuropathy **Multiomics** High throughput methods which analyse differences in DNA sequences, gene transcription and translation into polypeptides

Emerging therapeutic strategies	
Leber hereditary optic neuropathy	Gene therapy for ***MT-ND1*** LHON caused by m.3460G>A rAAV2-ND1 (NFS-02, Neurophth Inc., Huazhong University, Wuhan, China) Intravitreal injection to improve *MT-ND1* function via allotopic expression Currently in phase I/II clinical trial (NCT05820152) **MTS-AAV**: mitochondrial targeting sequence combined with an adeno-associated virus Alternative to allotopic expression to directly target the mitochondria Demonstrated improved visual outcomes in m.11778G>A mouse model (78) Similar success in *MT-ND6* mouse model (79)
Stem cell therapies	Intravitreal transplant of human iPSC-derived retinal ganglion cells in wild-type C57BL/6J mice (82) Enhanced delivery with **biomaterials**, which aim to recapitulate the interaction between cells and the extracellular matrix Phase I trial: two patients with end-stage age-related macular degeneration Subretinal transplant of retinal pigment epithelium with aid of human-vitronectin-coated polyester membrane Improved visual acuity during postoperative follow-up
Targeted gene editing	**Double-stranded DNA deaminase toxin A (DddA)** Originally a bacterial toxin expressed by *Burkholderia cepacia* Form of cytidine deaminase, which catalyses the conversion of C•G to T•A DddA-derived cytosine base editors (DdCBEs) specifically target mitochondria via a mitochondrial targeting sequence May be harnessed to treat m.14484T>C LHON pathogenic variant **Transcription activator-like effector nucleases (TALENs)** Harness endonucleases to cleave a target sequence from DNA Causes a shift in mitochondrial DNA heteroplasmy towards wild-type levels Pre-clinical proof of concept in m.14459G>A LHON with dystonia (LHOND) (87) **Zinc finger nucleases (ZFNs)** Again, aim to shift from heteroplasmy towards wild-type mitochondrial DNA levels Achieved by sequence-specific DNA binding zinc finger peptides linked to an endonuclease, which cause mtDNA breaks Experimental promise in field of mitochondrial diseases (88) Not applicable to homoplasmic mutations **Meganucleases** Aim to reduce mutant mitochondrial DNA Endonucleases target specific dsDNA sites M.5042C>T mouse model showed a reduction in mutant mitochondrial DNA (89) Not applicable to homoplasmic mutations
Neuroprotection	Ongoing trials are examining the role of **brain-derived neurotrophic factor** for improving retinal ganglion cell survival, especially in the context of glaucoma **Protrudin**, a scaffold molecule, may assist with retinal ganglion cell axon regeneration (93) **SOD2** and **SkQ1** may act to reduce production of reactive oxygen species to improve mitochondrial function in Leber hereditary optic neuropathy

The m.11778G>A *MT-ND4* mutation forms the therapeutic target for two further gene therapy constructs: scAAV2-P1*ND4*v2 (Bascom Palmer Eye Institute, FL, USA) and rAAV2-*ND4* (NFS-01, Neurophth Inc., Huazhong University, Wuhan, China). Both approaches use forms of an AAV2 vector to deliver a WT copy of the *MT-ND4* gene, again via allotopic expression following intravitreal injection (41, 42). scAAV2-P1*ND4*v2 has demonstrated expression of *MT-ND4* in non-human primates and a favourable safety profile in a mouse LHON model (43). A phase I dose-finding and safety trial (NCT02161380) found a favourable safety profile but the efficacy effect was likely small and not dose-related (44, 45). An initial human trial of rAAV2-*ND4* involved nine participants and demonstrated a significant improvement in BCVA of ≥0.3 logMAR in six patients. Long-term results confirmed the therapy’s acceptable safety profile. A subsequent multicenter clinical trial with 40 patients demonstrated a significant improvement in BCVA and, similarly, observed a bilateral improvement despite only the worse-seeing eye receiving an intravitreal injection (46–48). A phase III clinical trial examining the efficacy of rAAV2-*ND4* administered to LHON patients within 3 months of disease onset is underway (NCT03428178). Results are expected to be published in 2026.

Beyond gene therapy, idebenone is a ubiquinone analogue which bypasses dysfunctional complex I in the electron transport chain by delivering electrons directly to complex III. Idebenone has been approved by the European Medicines Agency for use in LHON in 2015 following results from the RHODOS and RHODOS-OFU trials, as well as an industry-sponsored expanded access programme, which demonstrated the effectiveness of idebenone in LHON patients treated within 1 year of symptom onset (49–51). The subsequent LEROS study established the long-term effectiveness of idebenone in LHON patients within 5 years after disease onset (52). Interestingly, the efficacy of idebenone varied depending on the causative mtDNA mutation, finding a possible worsening of visual outcomes in patients with the m.3460G>A mutation treated within the first year of vision loss.

Idebenone clinical trials demonstrate the importance of continuing treatment for at least 24 months to determine whether an individual will respond, with the treatment effect determined by the LHON disease phase and genotype. As with lenadogene nolparvovec, a meta-analysis showed superior visual outcomes in patients treated with idebenone relative to the disease’s natural history, with a *clinically relevant recovery* in BCVA from its lowest point. Other ubiquinone analogues are currently in phase I clinical trials for mitochondrial disorders, though not for IONs. For example, results from a phase I trial for KL1333 (NCT03888716) demonstrated a promising functional improvement in patients with primary mitochondrial diseases (53).

Lenadogene nolparvovec and idebenone offer hope for improving visual outcomes in LHON but are subject to limitations of clinical trial design. For example, using nadir to assess improvement in BCVA in trials may not be an accurate reflection of visual improvement given the subjective nature of measuring visual acuity, which may be influenced by poor effort or fixation. The lack of a control arm in gene therapy trials due to unexpected bilateral improvement and the need to compare with published natural history data also raises concerns about confounding variables.

The broader management of LHON focusses on reducing causes of increased ROS that have been implicated in LHON penetrance (54). Advising patients on smoking and alcohol consumption already forms a key part of the clinical consultation. Other medications may also cause mitochondrial toxicity although it is difficult to prove direct causality. For example, some antibiotics designed to target the bacterial ribosome may cross-react with mitochondrial machinery, and these have been linked with development of the LHON phenotype (55).

## DOA therapeutic opportunities

**Figure 2:**
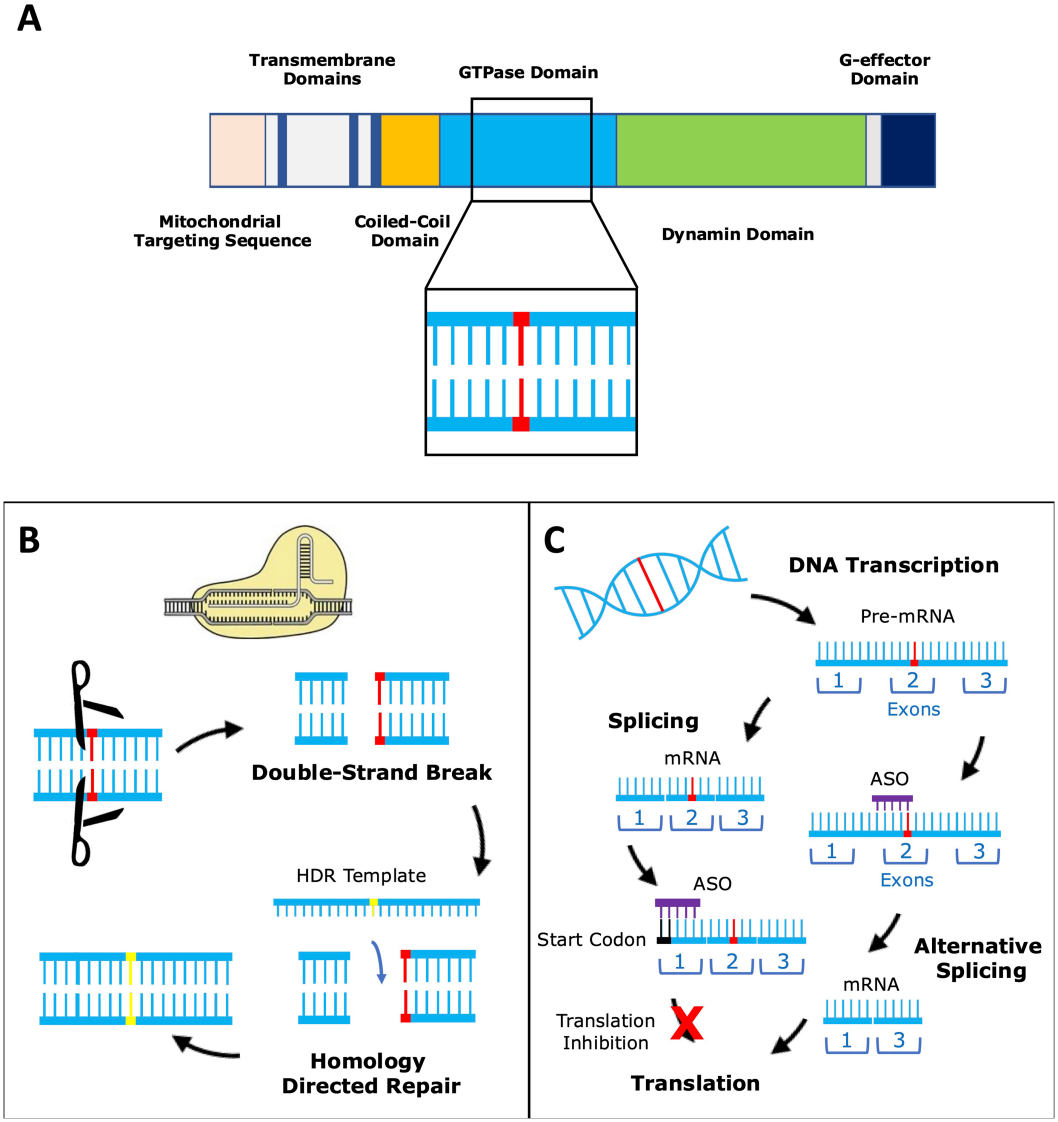
Reproduced with permission from Chen et al. (2023). **(A)** The *OPA1* gene codes for a large multimeric GTPase that localises to the mitochondrial inner membrane and regulates a number of important mitochondrial functions. Over 500 pathogenic variants (represented by the red base pair) of *OPA1* have been reported and these span the entire length of the gene. **(B)** *OPA1* variants can be corrected by gene editing using CRISPR-Cas9. Double-strand breaks in DNA are introduced by Cas9 and the correct base sequence is inserted by homology directed repair (HDR) using an HDR template. **(C)** Another strategy is to modify *OPA1* expression by altering gene transcription using antisense oligonucleotides (ASOs). ASOs can bind to mRNA near the translation start site and either enhance or suppress translation. Alternatively, the ASO can bind to pre-mRNA resulting in alternative splicing, which could be used to restore normal splicing in the setting of *OPA1* splice variants.

There are no approved disease-modifying treatments in patients with DOA, for whom the mainstay of therapy is supportive management. In parallel to the developments occurring in the therapeutic pipeline for LHON, there has been interest in gene therapies for DOA—but unlike gene therapies targeting m.11778G>A *MT-ND4*, trials for DOA are at an earlier stage of translational development. DOA is a genetically heterogeneous condition, with more than 500 reported pathogenic mutations, most of which cause haploinsufficiency, but with an important subgroup exerting a dominant-negative effect. Rather than attempting to resolve each mutation individually, antisense oligonucleotide (ASO) technology can improve *OPA1* expression in a mutation-agnostic fashion. Emerging therapies must address haploinsufficiency as the predominant causative mechanism in DOA, for which there are two constructs entering early phase I trials: STK-002 from Stoke Therapeutics (ISRCTN41725621) (56) and PYC-001 from PYC Therapeutics (NCT06461286) (57). Both harness an ASO approach to upregulate reduced *OPA1* gene expression arising from haploinsufficiency (Figure 2). Experimental cell models of DOA transfected with STK-002 and PYC-001 have, respectively, shown an increase in OPA1 protein—a result corroborated by rabbit and non-human primate models.

Another strategy being explored is mRNA *trans*-splicing, based on mRNA correction, which harnesses the cell’s natural ability for splicing reactions using the spliceosome (58). This introduces a therapeutic effect by splicing the mutated endogenous mRNA transcript and correcting it with a WT sequence. Importantly, this technology works independently of the causative pathogenic mutation, does not risk *OPA1* over-expression and could be applied to disease-causing variants causing a dominant-negative effect. Neither does *OPA1 trans*-splicing alter the alternatively spliced exons 4, 4b and 5b, therefore preserving physiological expression of the eight OPA1 isoforms in humans.

Idebenone use in DOA remains an area of investigation, complicated by the disease’s slow progression and uncertain natural history. It is hoped that idebenone may restore mitochondrial function and hence, prevent RGC loss in DOA due to its ability to bypass dysfunctional respiratory complex I. Initial studies were low-powered, but showed a positive effect of idebenone on vision (59, 60) and these have been followed by a recent prospective phase II trial, which showed a small degree of visual recovery with a corresponding improvement in vision-related quality of life (61). The authors stress uncertainty surrounding this observed result, and question whether visual recovery is truly due to treatment effect, placebo or natural history. In the absence of other disease-modifying treatments, idebenone is being used ‘off-label’ in DOA by some clinicians.

As in LHON, salvage of RGC degeneration may be approached holistically by reducing production of ROS or addressing the risk of mitochondrial toxicity. Lifestyle modifications may include reducing patients’ exposure to low wavelength visible blue light via a filter (62), while exposure to near-infrared light therapy has shown improved mitochondrial function in models of retinal damage (63). Vitamin B3 and NAD^+^ precursors such as nicotinamide have been investigated for their role in mitochondrial function in mitochondrial diseases more generally (64). Upregulating mitochondrial biogenesis using supplements to target their metabolic pathways shows experimental benefit in mitochondrial function, but further work is needed to determine their efficacy, including their ability to cross the blood–brain barrier and reach RGCs.

## Disease modelling approaches

The burden of IONs is laid bare by a paucity of approved disease-modifying treatments. A key to circumventing this is the development of accurate disease models to recapitulate unique pathological mechanisms, such as preferential RGC degeneration, to identify biomarkers. It follows that with improved disease modelling comes opportunities for therapeutic targets. This is particularly challenging in the case of IONs due to inherent difficulties procuring human retinal or optic nerve tissue. A mouse animal model is limited due to the absence of a papillomacular bundle, different proportions of RGC subtypes (65) and, in the case of DOA, only four *OPA1* splice isoforms rather than eight (66).

These difficulties are overcome with induced pluripotent stem cells (iPSCs), somatic cells which are converted into embryonic stem cell-like cells, and then encouraged to differentiate into a cell of interest (67). iPSCs are capable of differentiating into any cell type of the three germ layers and, beyond acting as a model to reproduce the pathological processes in IONs for study *in vitro*, also allow an assessment of cells derived from affected carriers. In the case of LHON, for example, differentiation of iPSCs into RGCs with a homoplasmic double mtDNA variant affecting *MT-ND1* and *MT-ND6* demonstrated significantly increased rates of apoptosis compared with control cell lines (68).

Recent iPSC-RGC modelling is continuing to reveal the multifaceted nature of the *OPA1* gene and its contribution to the development of DOA. Generation of a cell line expressing the R445H mutation causing *DOA plus* demonstrated new findings relating to calcium homeostasis, with implications for the pathogenesis of DOA more generally (69). The study demonstrated defects in intracellular calcium buffering, likely related to the physiological role of calcium in maintaining mitochondrial membrane potential. Research in this novel area of DOA pathogenesis is ongoing, and it will be interesting to characterise the specific impact of other *OPA1* mutations on intracellular calcium trafficking.

iPSC models are also an attractive method for assessing possible therapeutic targets in genetic causes of vision loss. They may be combined with clustered regularly interspaced short palindromic repeats-Cas9 (CRISPR-Cas9), which edit nuclear-encoded gene mutations using Cas9, an endonuclease, to cause a double-stranded DNA (dsDNA) break. In a patient-derived iPSC model of DOA, a CRISPR-Cas9 correction of the c.1334G>A mutation restored mitochondrial homeostasis to ameliorate a pathological phenotype (70). A similar proof of concept approach was reported in cells expressing *ABCA4* variants derived from patients with Stargardt disease, which were corrected via CRISPR-Cas9 with no off-target effects (71). It is hoped that CRISPR-Cas9 corrected cells might be used for retinal transplants which, in the case of autologous iPSCs, circumvents the challenges presented by immunogenicity and graft rejection. However, significant challenges will need to be overcome, including how to transplant the differentiated RGCs, ensure that the right complement of RGC subtypes are replaced, ensure their survival, and importantly, how they establish the right connection in a retinotopic pattern (72).

Excitingly, new technology is broadening our view of causative mutations leading to IONs. High throughput sequencing can detect hitherto unknown differences in structural variants and has increased the number of genes and variants implicated in heritable diseases, redefining the clinical detection of IONs. Revealing genetic heterogeneity leads to an expansion of a disease’s genotype–phenotype landscape and as a result, it is argued that classification should now shift away from eponymy towards a description of the direct effect of the mutation on protein function (27). Precisely defining disparate pathological processes affecting IONs, which include haploinsufficiency, gain-of-function and dominant-negative mechanisms, facilitates the refinement of targeted therapeutic approaches.

**Figure 3:**
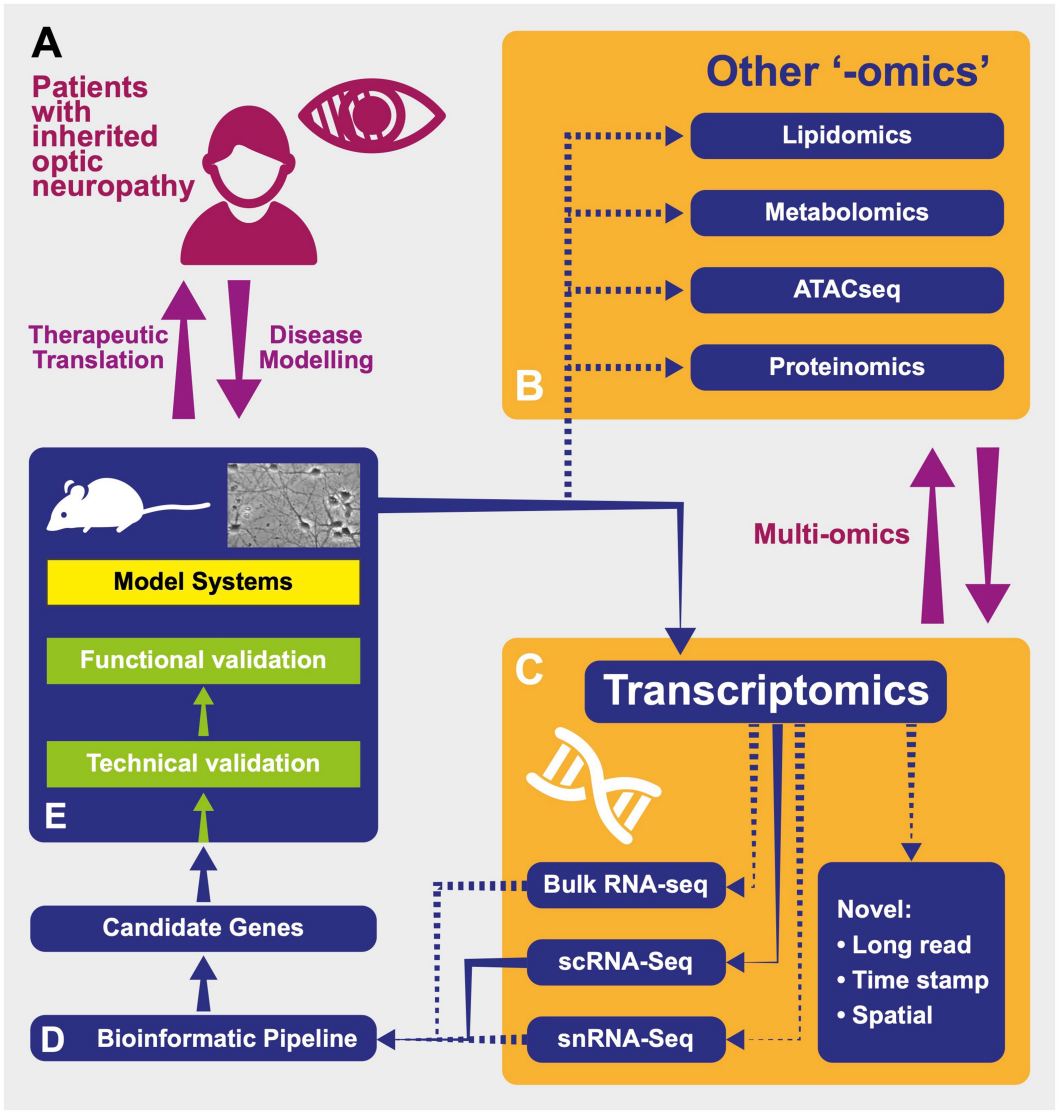
Reproduced with permission from Gilhooley et al. (74). A schematic representing the processes of modelling and investigating inherited optic neuropathies using “-omics” methods. **(A)** Model diseases such as DOA and LHON can be powerful tools in which to investigate the effects of mitochondrial dysfunction. The study of patients with inherited optic neuropathies is often a two-way process: in one direction, the characterisation of their phenotype and genotype allows the development of useful disease models (for example, cell- and animal-based). These provide an efficient environment in which to increase our understanding of underlying disease processes as well as a testing ground for novel therapies before their return in the opposite direction, back into patients. **(B)** Tissues from model systems can be investigated using multiple “-omics” techniques—in some cases, these can be performed simultaneously and synthesised into a multiomic approach. ATACseq—Assay for Transposase Accessible Chromatin—a method for assessing which areas of the chromatin superstructure are open and so likely available for active in transcription, is an example of the rapidly expanding field of *epigenomics*. **(C)** Multiple transcriptomic techniques are available and have particular strength in different experimental situations. “Novel”: Novel techniques are emerging that will further the resolution of these techniques including those with abilities to sequence longer transcripts in one read and those that integrate temporal and spatial information regarding transcripts (see text). RNA-seq—RNA-sequencing; scRNA-seq—single-cell RNAseq; snRNAseq—single nucleus RNAseq. **(D)** Transcriptomic techniques gain power from the large quantity of data that they produce. This necessitates adequately designed bioinformatic pipelines that are tailored to the exact scientific question being asked in order to produce a list of candidate genes for further investigation back in model systems. **(E)** Model systems of diseases can include those based on cultured patient cells or be animals carrying pathogenic variants, leading to phenocopies of human disease. Samples from these models used in transcriptomic analysis can include tissue (such as retinas) or cell cultures.

In addition to an expanding understanding of ION genetic determinants, next-generation sequencing (NGS) promises to identify new genomic variants and elucidate questions surrounding disease pathology, such as incomplete penetrance in LHON. This is heralded by “-omics” technologies, which constitute a set of high throughput methods to analyse differences in, among others, DNA sequences, gene transcription and translation into polypeptides (73, 74). These techniques, respectively, comprise genomics, transcriptomics and proteomics (Figure 3).

Transcriptomics, the study of all RNA transcribed within a cell, is an especially promising approach for defining pathological processes in IONs. This is achieved by RNAseq, in which high throughput technology directly sequences cDNA to identify genetic variants not detected at the genomic level by established gene expression microarrays. These include the ability to measure rare transcripts, splice variants and non-coding transcripts (75). RNAseq has been successfully deployed in fields beyond the IONs including breast cancer, where patients negative for BRCA1 transcripts by conventional genome sequencing have been shown to express novel pathogenic splice variants of BRCA1 (76). The advent of single cell RNAseq has given rise to spatial transcriptomics, a study of cells within their positional context, which promises to correlate morphological and transcriptomic information to further resolve understanding of affected cells in IONs (74, 77).

Transcriptomics is increasingly integrated into a multiomic approach, which synthesises analysis from several high throughput screening technologies to identify new biomarkers. The transition of this approach from the research environment to a clinical one signifies an expanding era of precision medicine, in which treatments are developed in response to factors influencing individuals’ gene expression and protein production. The scope for improved diagnostic capability is particularly marked for those patients who do not yet demonstrate a confirmed molecular diagnosis, but for whom a definitive diagnosis is a requisite for therapeutic options, as with voretigene neparvovec-rzylv (Luxturna) for treating biallelic *RPE65-*associated retinal dystrophy (78).

Recent years have seen an improvement in access to sequencing technologies in the clinical environment, with increased availability and reduced costs. The advent of testing ‘panels’ for suspected diagnoses has helped refine the diagnostic process, while genetic counsellors assist patients with important discussions regarding family planning and psychosocial adjustment. However, pathophysiological questions remain and a large proportion of patients still do not have a confirmed molecular diagnosis, which limits genetic counselling and access to research studies. The technological developments seen in NGS and the possibilities this creates for precision medicine may help with this unmet need and improve the outlook for patients with IONs.

## Towards new biomarkers and therapeutic targets

An allotopic expression gene therapy approach is currently being considered for *MT*-*ND1* LHON caused by m.3460G>A (NCT05820152). Alternative LHON gene therapies employ an MTS combined with an AAV (MTS-AAV), rather than allotopic expression, to directly target mitochondria. This has been tested in a mouse model of LHON, in which an MTS-AAV was used to deliver a WT copy of the *MT*-*ND4* gene. The transcript was successfully incorporated into the mitochondria, as observed in cells with the m.11778G>A mutation, and successfully reduced visual loss and optic atrophy in mice (79). This approach has more recently shown promise with successful delivery of *MT*-*ND6* to the mitochondrial genome in mice (80), which is of particular interest because there are currently no trials assessing LHON therapies caused by *MT*-*ND6* mutations.

Beyond the three common LHON mtDNA mutations, NGS has identified rare genetic variants associated with the LHON phenotype, offering scope for future therapeutic targets and refined genetic counselling. *DNAJC30* is a gene recently implicated in LHON, which produces a protein localised to the inner mitochondrial membrane to assist with complex I repair mechanisms and ATP synthesis. Biallelic mutation of *DNAJC30* c.152. A>G causes autosomal recessive LHON (OMIM #619382). The clinical presentation tends to comprise an earlier age of onset, often with bilateral eye involvement and a higher rate of spontaneous visual recovery. Importantly, idebenone has been reported to significantly improve vision in LHON caused by *DNAJC30* (81). ‘Susceptibility’ alleles that increase the risk of vision loss in people who carry a LHON mutation may also offer scope for future therapeutic targets. *PRICKLE3*, which physiologically influences ATP synthase function, increases the risk of vision loss in people with the m.11778G>A mutation. Whole-exome sequencing on a large cohort of LHON patients identified a novel locus located at Xq25-27.2 in three families. The same study demonstrated reduced ATP production and subsequent RGC degeneration in a *Prickle-3* knock-out mouse model (82).

Stem cell therapies have also been the focus of several preclinical and clinical studies. Successful proof of concept studies have shown that human iPSC-derived RGCs can be intravitreally transplanted in WT C57BL/6J mice—the transplanted RGCs integrate within the retinal ganglion cell layer of host retina and exhibit electrophysiological function similar to native mouse RGCs (83). Such therapeutic promise of iPSCs, and pluripotent stem cells more generally, is being optimised with biomaterials which may assist with all stages of stem cell generation from bench to bedside (84). This is achieved by *dynamic reciprocity* and tissue-specific *tensional homeostasis*—two concepts which characterise the interaction between stem cells and biomaterials to recreate the extracellular matrix (ECM) (85). Biomaterials—specifically decellularized extracellular matrix, synthetic polymers and natural hydrogels—in conjunction with iPSCs are in the early stages of investigation for RGC replacement in IONs (84). It is hoped that biomaterials will aid a more accurate recapitulation of the interaction between cells and their ECM. More broadly, a phase I study investigated transplanting a retinal pigment epithelium patch derived from human embryonic stem cells aided by a human-vitronectin-coated polyester membrane. The transplant was placed into the subretinal space of two patients with end-stage age-related macular degeneration, both of whom experienced an improvement in BCVA following the procedure (86).

Targeted gene editing technologies are being investigated as an alternative means of therapy in mitochondrial diseases. Double-stranded DNA deaminase toxin A (DddA) is a bacterial toxin expressed by *Burkholderia cepacia,* and has been harnessed as a CRISPR-free RNA-free means of precision genome editing. DddAs are a form of cytidine deaminase, which catalyse the conversion of C•G to T•A and, as such, may offer a means of treating the LHON-causing m.14484T>C variant (87). DddA-derived cytosine base editors (DdCBEs) are a mitochondrial specific form of DddA, which contain an MTS to edit bases within the mitochondrial genome. Their ability to cause targeted mutations has been tested on several mitochondrial genes *in vitro* including *MT-ND1* and *MT-ND4* with efficiencies up to 50% (66).

Other methods of editing the mitochondrial genome are in early stages of experimental development and, as with CRISPR-Cas9, employ endonucleases to edit a DNA transcript. Transcription activator-like effector nucleases (TALENs) specifically cleave a target sequence from DNA to cause a shift in mtDNA heteroplasmy towards WT mtDNA levels, achieved by fusing a transcription activator-like domain to a DNA cleavage domain—usually a Fokl endonuclease. This has been experimentally tested using a TALEN designed to target the m.14459G>A mtDNA point mutation causing LHON with dystonia (LHOND). Subsequent analysis showed a reduction in the amount of mtDNA containing the m.14459G>A mutation (88).

Zinc finger nucleases (ZFNs) and meganucleases again aim to reduce heteroplasmy and restore WT mtDNA levels. In the case of ZFNs, this is achieved by sequence-specific DNA binding zinc finger peptides that are linked to an endonuclease to bring about mtDNA breaks. This technique has shown experimental promise in mitochondrial diseases, where cells with the m.8993T>G mutation demonstrated a shift away from heteroplasmy towards WT mtDNA, corroborated by an improvement in mitochondrial function (89). Meganucleases use an endonuclease to bring about site-specific dsDNA breaks. This was tested in the m.5042C>T mouse model, delivered by AAV9, and experimentally demonstrated a reduction in mutant mtDNA (90). These techniques cannot, however, be used in individuals with a homoplasmic mutation, as is the case for most LHON carriers—a benefit of the approach using DdCBEs.

Neuroprotective strategies provide an approach to therapeutics for IONs and, importantly, act independently from the causative mutation. Brain-derived neurotrophic factor is an established agent in neurodegenerative disease and is currently being investigated for improving RGC survival, particularly in glaucoma (91–93). Protrudin, a scaffold molecule associated with the endoplasmic reticulum has been assessed as a candidate to assist with axon regeneration. Importantly, experimental overexpression of Protrudin caused regeneration of RGC axons in an injured mouse optic nerve following a simulated crush injury (94). Neuroprotection may also be harnessed to reduce ROS as a means of circumventing apoptosis in RGCs. Experimental evidence in patient-derived fibroblasts supports the use of SOD2 and SkQ1, agents which act to reduce ROS and downregulate pro-inflammatory pathways, to ameliorate mitochondrial dysfunction in LHON (95, 96). It is foreseen that transcriptomics will aid the identification of new biomarkers to further refine neuroprotective strategies in the coming years (74, 97).

## Conclusion

Improved disease models and sequencing techniques are expanding our knowledge of the causative genes in IONs and their variable phenotypes. In this review, we have assessed how these new technologies are bridging the gap between disease mechanisms and complex development of a phenotype, which cannot simply be explained by the disease-causing genetic variant. There is an unmet clinical need, and new therapeutic approaches are required to improve the visual outcome for these important causes of blindness in children and young adults.

## Footnotes

^1^ https://www.lovd.nl/OPA1

## Glossary

AAV: Adeno-associated virus
AAV2: Adeno-associated virus serotype 2
AAV9: Adeno-associated virus serotype 9
ASO: Antisense oligonucleotide
ATP: Adenosine triphosphate
BCVA: Best-corrected visual acuity
CRISPR: Clustered regularly interspaced short palindromic repeats
DddA: Double-stranded DNA deaminase toxin A
DdCBE: DddA-derived cytosine base editor
DOA: Dominant optic atrophy
dsDNA: Double-stranded DNA
ECM: Extracellular matrix
GDP: Guanosine diphosphate
GTP: Guanosine triphosphate
ION: Inherited optic neuropathy
ipRGC: Intrinsically photosensitive retinal ganglion cell
iPSCs: Induced pluripotent stem cells
LHON: Leber hereditary optic neuropathy
LHOND: Leber hereditary optic neuropathy with dystonia
L-OPA1: Long form of OPA1 protein
MS: Multiple sclerosis
mRNA: Messenger RNA
mtDNA: Mitochondrial DNA
MTS: Mitochondrial targeting sequence
MTS-AAV: Mitochondrial targeting sequence combined with an adeno-associated virus
NAD: Nicotinamide adenine dinucleotide
NGS: Next-generation sequencing
NMD: Nonsense mediated decay
OCT: Optical coherence tomography
RGC: Retinal ganglion cell
rAAV: Recombinant adeno-associated virus
RNFL: Retinal nerve fiber layer
ROS: Reactive oxygen species
S-OPA1: Short form of OPA1 protein
scAAV: Self-complementary adeno-associated virus
TALEN: Transcription activator-like effector nuclease
WT: Wild type
ZFN: Zinc finger nuclease
